# Fetal Kidney Length as a Parameter for Determining Gestational Age From the 20th Week in Healthy Women With Uncomplicated Pregnancy

**DOI:** 10.7759/cureus.108682

**Published:** 2026-05-11

**Authors:** Guljhari Lal, Tejas Patel, Gurjinder S Bajwa, Ankit Jakhar, Dinesh K Panda

**Affiliations:** 1 Department of Radiology, JIET Medical College and Hospital, Jodhpur, IND; 2 Department of Radiology, RadSquare Imaging Centre, Ahmedabad, IND; 3 Department of Radiology, Max Super Specialty Hospital, Mohali, Mohali, IND

**Keywords:** fetal biometry, fetal kidney length, gestational age, obstetrics, radiology, ultrasound

## Abstract

Background: For the best obstetric care, gestational age (GA) must be accurately estimated. However, as biological variability increases in the second and third trimesters, the accuracy of traditional biometric markers decreases. In this regard, fetal kidney length (FKL) has become a viable substitute due to its steady linear growth and its independence from growth disorders. The purpose of this study was to determine the function of FKL in determining GA beyond 20 weeks and to examine its association with recognised fetal biometric parameters.

Aim: The study aims to evaluate FKL as a parameter for determining GA from 20th weeks onwards in healthy women with uncomplicated pregnancy.

Methods: In this prospective cross-sectional study, conducted between April 2023 and March 2024, at the department of radio-diagnosis at a tertiary care facility, 203 pregnant women with clear-cut singleton pregnancies longer than 20 weeks of gestation participated. Using the most recent menstrual cycle and, if available, an early ultrasound, GA was calculated. Bi-parietal diameter, head circumference, abdominal circumference, femur length, and FKL were all measured using ultrasonography. Relationships between FKL GA and other biometric markers were evaluated using Pearson's correlation coefficient.

Results: GA and FKL showed a significant positive connection (r = 0.953, p < 0.001). FKL was also shown to be significantly correlated with head circumference (r = 0.948), femur length (r = 0.887), biparietal diameter (r = 0.949), and abdomen circumference (r = 0.954). According to regression analysis, a 1 mm increase in FKL was associated with a GA increase of almost 0.9 weeks.

Conclusion: FKL can be used as a useful supplement to regular fetal biometry and is a dependable and repeatable measure for determining GA after 20 weeks.

Clinical implications: The accuracy of gestational dates can be increased by including FKL into regular ultrasonography, especially in late-presenting foetuses with unclear menstrual histories.

## Introduction

Prenatal screening, fetal growth tracking and delivery scheduling all depend on an accurate assessment of gestational age (GA), which is essential for obstetric care [1]. Crown-rump length (CRL), which exhibits little biological variability, can be used to accurately determine GA in early pregnancy [2]. However, due to growing inter-individual variability in fetal growth, routinely used biometric markers such as bi-parietal diameter (BPD), head circumference (HC), abdominal circumference (AC) and femur length (FL) gradually lose accuracy in the second and third trimesters [3]. This limitation is significant in clinical settings where women have uncertain last menstrual period (LMP) dates or arrive late for prenatal treatment.

Alternative sonographic factors have been investigated to improve GA estimation in later pregnancy as a solution to this problem. Fetal kidney length (FKL) is one of these that has shown promise as a trustworthy marker [4]. Throughout pregnancy, the fetal kidneys show a steady linear growth pattern. Moreover, the clinical research indicates that they grow by about 1-2 mm every two weeks [5]. Renal length is a potentially stable characteristic even in situations like intrauterine growth restriction, which is significant because it seems to be mostly unaffected by fetal growth abnormalities [6].

Previous research has shown that FKL and GA are strongly correlated, with correlation coefficients that are on par with or higher than those of traditional biometric markers [7]. Additionally, it is theoretically possible to assess FKL during regular obstetric ultrasonography, and it is simple to include into standard fetal biometry methods without incurring substantial time or expense burdens [8].

A significant percentage of pregnant women present for prenatal treatment in the second or third trimester with an unclear or inconsistent menstrual history in many real-world clinical settings, particularly in low- and middle-income countries [9]. In these situations, erroneous GA calculation may result in less-than-ideal treatment choices, such as improper birth scheduling, incorrect fetal growth restriction diagnosis and elevated postnatal morbidity [10]. There are important therapeutic ramifications to the identification of a straightforward, repeatable and trustworthy sonographic metric like FKL.

FKL has the potential to increase the accuracy of gestational dating in situations when traditional parameters are constrained by providing a measurement that shows steady growth and less susceptibility to growth-related fluctuation [11]. By incorporating FKL into standard obstetric ultrasonography, pregnancy care might be optimised, diagnostic confidence could be increased, and maternal and newborn outcomes could be improved. Because of these benefits, FKL may be a useful supplementary or adjunct measure for determining GA throughout the second and third trimesters. The present study aims to evaluate the correlation between FKL and GA and assess its utility as an adjunct parameter for GA estimation after 20 weeks in healthy women with uncomplicated singleton pregnancies, and to assess its correlation with established biometric parameters.

## Materials and methods

This prospective cross-sectional study was conducted in the department of radiodiagnosis at a tertiary care centre over a period of 12 months (April 2023-March 2024) following institutional ethics approval. A total of 203 pregnant women with uncomplicated singleton pregnancies beyond 20 weeks of gestation were included. Only women with a reliable LMP and regular menstrual cycles were enrolled, and GA was determined using a reliable LMP and confirmed by early ultrasonography whenever available. Patients with multiple gestations, fetal anomalies, intrauterine growth restriction, oligohydramnios, polyhydramnios, or maternal comorbidities such as diabetes and hypertension were excluded to minimise confounding factors.

All participants underwent standardised obstetric ultrasonography using GE Voluson P8 (GE HealthCare, Chicago, IL, USA) and Philips Affiniti 70 (Philips Healthcare, Amsterdam, Netherlands) ultrasound systems equipped with 3.5 MHz convex transducers. Ultrasonography was performed with the patient in the supine position. Measurements were performed by radiologists with experience in fetal ultrasonography using standardised protocols. Fetal biometric parameters, including BPD, HC, AC, and FL, were measured according to established guidelines. FKL was assessed in the sagittal plane by visualising the full longitudinal extent of either kidney, ensuring exclusion of the adrenal gland, and measuring the maximum pole-to-pole length. Measurements were obtained from either kidney, and whenever both kidneys were adequately visualised, the mean renal length was considered for analysis. Each measurement was obtained three times, and the mean value was recorded to improve accuracy. GA was primarily determined based on LMP and correlated with sonographic parameters.

Statistical analysis was performed using IBM SPSS Statistics for Windows, Version 30 (Released 2024; IBM Corp., Armonk, New York, United States), with continuous variables expressed as mean ± standard deviation. Pearson’s correlation coefficient was used to assess the relationship between FKL and GA, as well as with other biometric indices, with statistical significance set at p ≤ 0.05.

## Results

The study included 203 pregnant women with a mean age of 26.46 (SD = 3.47) years. Out of 203 patients, 100 (49.3%) were in their second trimester with a mean age of 26.26 (SD = 3.18) years, and 103 (50.7%) were in their third trimester with a mean age of 26.27 (SD = 3.75) years.

**Figure 1:**
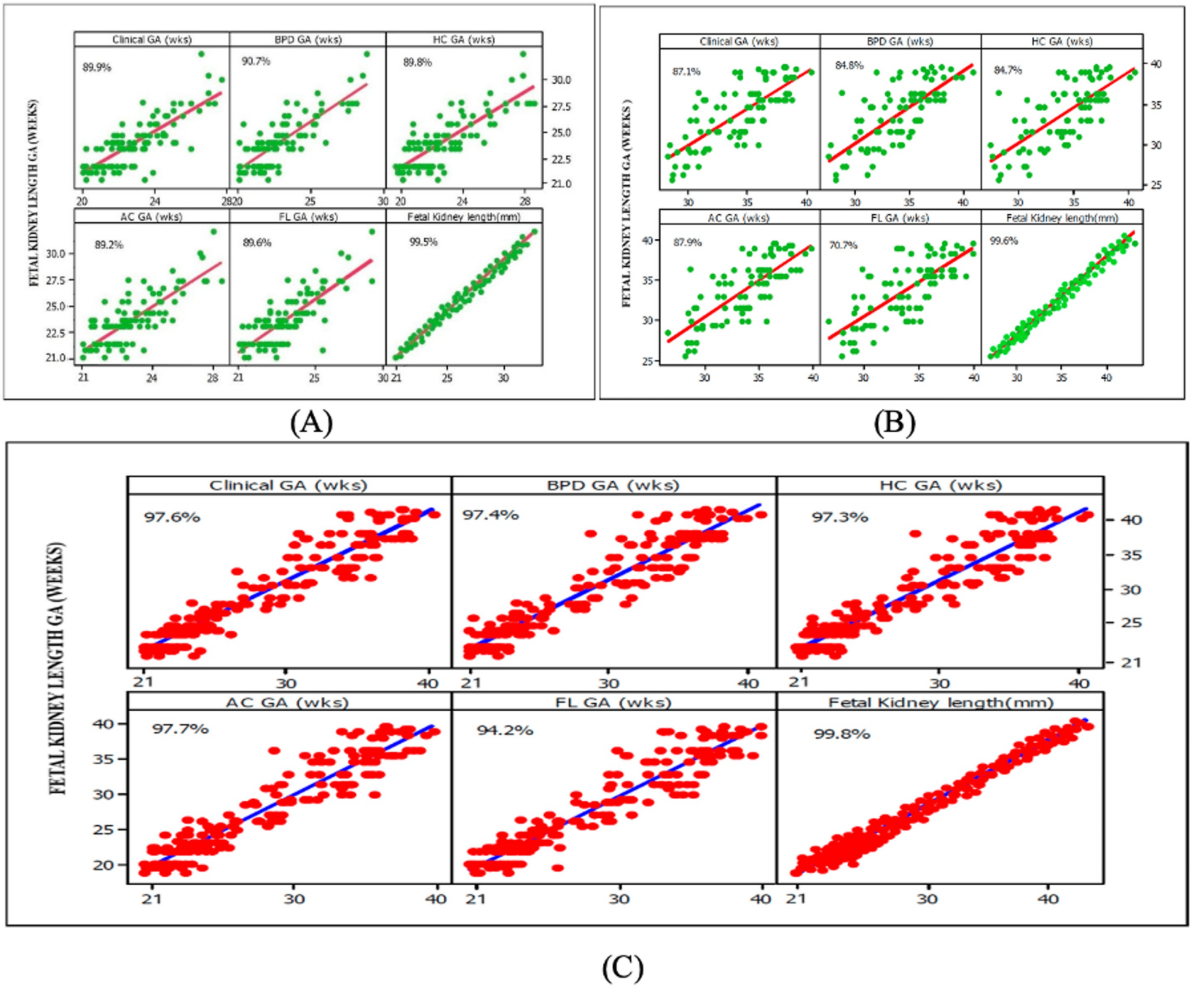
Correlation and regression analysis between FKL GA, and fetal measurements. (A) Second trimester; (B) third trimester; (C) overall analysis. FKL GA: fetal kidney length gestational age; BPD: biparietal diameter; HC: head circumference; AC: abdominal circumference; FL: femur length

Figure 1 shows that FKL GA and fetal measurements are strongly positively correlated in patients in the second and third trimesters, as well as in all patients.

The following regression model was derived from the aforementioned analysis: GA (weeks) = 2.1 + 0.92 * FKL (mm). Therefore, it may be concluded that there is roughly a 0.9-week increase in GA for every 1 mm increase in FKL.

FKL GA and fetal measures are related, as Table 1 illustrates. There was statistical significance in every correlation. FKL GA had the lowest association with AC (r = 0.797) and the highest correlation with BPD (r = 0.823) for patients in the second trimester. FKL GA had the lowest connection with FL (r = 0.491) and the highest correlation with AC (r = 0.774) for patients in the third trimester. Overall, FKL GA's correlation was lowest with FL (r = 0.887) and highest with AC and CGA (r = 0.954 and 0.953, respectively).

**Table 1:** Association between FKL GA and fetal measurements. FKL GA: fetal kidney length gestational age; CGA: clinical gestational age; BPD: biparietal diameter; HC: head circumference; AC: abdominal circumference; FL: femur length; R-coeff.: Pearson correlation coefficient

Pair	Second Trimester		Third Trimester		Overall	
	R-coeff.	p-value	R-coeff.	p-value	R-coeff.	p-value
FKL GA vs. CGA	0.809	<0.001	0.758	<0.001	0.953	<0.001
FKL GA vs. BPD	0.823	<0.001	0.720	<0.001	0.949	<0.001
FKL GA vs. HC	0.807	<0.001	0.718	<0.001	0.948	<0.001
FKL GA vs. AC	0.797	<0.001	0.774	<0.001	0.954	<0.001
FKL GA vs. FL	0.802	<0.001	0.491	<0.001	0.887	<0.001

Table 2 provides information related to the correlation between fetal measures and CGA. There was statistical significance in every correlation. CGA had the highest connection with HC (r = 0.863) and the lowest correlation with FKL GA (r = 0.809) during the second trimester. In the third trimester, CGA had the lowest connection with FL (r = 0.748) and the highest correlation with HC (r = 0.955). Overall, there was little connection between clinical gestational age (CGA) and FL (r = 0.939) and nearly equal correlations with BPD, HC, and AC (r = 0.986, 0.987, and 0.985, respectively).

**Table 2:** Association between CGA and fetal measurements. FKL GA: fetal kidney length gestational age; CGA: clinical gestational age; BPD: biparietal diameter; HC: head circumference; AC: abdominal circumference; FL: femur length; R-coeff.: Pearson correlation coefficient

Pair	Second Trimester		Third Trimester		Overall	
	R-coeff.	p-value	R-coeff.	p-value	R-coeff.	p-value
CGA vs. BPD	0.861	<0.001	0.945	<0.001	0.986	<0.001
CGA vs. HC	0.863	<0.001	0.955	<0.001	0.987	<0.001
CGA vs. AC	0.837	<0.001	0.951	<0.001	0.985	<0.001
CGA vs. FL	0.841	<0.001	0.748	<0.001	0.939	<0.001
CGA vs. FKL GA	0.809	<0.001	0.758	<0.001	0.953	<0.001

Table 3 demonstrates a progressive and nearly linear increase in mean FKL with advancing GA, indicating a consistent growth pattern throughout the second and third trimesters. This steady increment supports the strong positive correlation observed between FKL and GA.

**Table 3:** Mean fetal kidney length according to gestational age.

Gestational Age (Weeks)	Mean FKL (mm)
21	21.6
24	24.8
28	29.5
32	33.9
36	38.0
40	43.0

## Discussion

The current study shows that FKL and GA have a strong linear link, with a correlation coefficient that is close to 0.9, indicating a very high degree of association. This result is in line with new research that indicates FKL is a trustworthy measure for estimating GA during the second and third trimesters, especially when the accuracy of traditional biometric indices declines.

The idea that FKL consistently increases linearly with increasing gestation is supported by a recent study by Tulasi et al. (2025) [12], which found an even larger association (r = 0.975, p < 0.001). Their investigation also showed that FKL grows proportionately across gestational weeks, confirming its usefulness as a reliable sonographic marker, which is consistent with our findings. Similarly, according to a Cureus-based study by Kumar et al. (2025) [13], FKL measures closely match gestational milestones (e.g., 26 mm at 26 weeks and 35 mm at 35 weeks), further supporting the linear growth pattern seen in our dataset.

Baskaran et al. (2025) [14] observed that FKL grows proportionately with GA and may estimate GA with a reasonably low standard error (±10.32 days), which is consistent with our results. Crucially, their research highlighted that FKL becomes more helpful later in pregnancy, when the precision of conventional measures decreases. This observation is in line with our results, which showed that FKL remained highly correlated even throughout the third trimester, confirming its function as a stable metric that is unaffected by rising biological variability.

Our investigation showed similar relationships between FKL and other biometric indices, including BPD, HC, AC and FL. According to current research, FKL performs at least as well as standard metrics and may even outperform them in certain clinical situations [15]. Additionally, research has shown that renal length is less impacted by fetal growth abnormalities than other metrics, which preserves its accuracy in situations when parameters like AC and FL may change.

A substantial positive association between mean kidney length and GA was also found in a study by Shrivastava and Laddad (2023) [16] in an Indian population, with an R^2^ value of 0.769, that showing good prediction ability. This bolsters the claim that FKL can function as a reliable solo or adjunct parameter and validates our regression results. Thus, FKL demonstrated a strong correlation with GA and may serve as a valuable complementary parameter alongside conventional fetal biometry. Furthermore, other recent research has shown that FKL measurement is technically straightforward, repeatable and easily included in standard obstetric ultrasonography without incurring extra expenses or time commitments [17].

Despite these benefits, the majority of research, including the current study, indicates that to optimise accuracy, FKL should ideally be combined with other biometric factors. A multi-parametric technique improves the robustness of GA prediction and helps account for individual heterogeneity, especially in late-presenting pregnancies. Overall, the results of this study support the clinical usefulness of FKL as a dependable and useful measure for estimating GA in the second and third trimesters and are in good agreement with recent literature.

Limitations

Generalisability may be limited by the fact that this study was carried out at a single location with a comparatively homogeneous population. The present study was cross-sectional rather than longitudinal; therefore, interval growth assessment of FKL could not be evaluated. FKL measuring interobserver variability was not evaluated, which may influence the reproducibility of FKL measurements despite the use of standardised ultrasonographic techniques and repeated measurements. Furthermore, the exclusion of high-risk pregnancies limited its application in pathological circumstances. To assess intra-individual growth patterns, longitudinal follow-up was not carried out. Agreement analysis, such as Bland-Altman analysis, was not performed and may be explored in future studies to further evaluate concordance between FKL and conventional biometric parameters. Although the present study was limited to uncomplicated pregnancies, FKL may also have potential utility in high-risk pregnancies where conventional biometric parameters are affected by abnormal growth patterns. Technical difficulties in visualisation and accurate measurement of FKL may occur in certain fetal positions, particularly occipitoposterior positions. Furthermore, foetuses with renal anomalies such as hydronephrosis were excluded from the study, which may limit the applicability of the findings in pathological renal conditions.

## Conclusions

For determining GA in the second and third trimesters, FKL shows promise as a clinically useful adjunct metric. It is especially helpful in cases with ambiguous dates and late-presenting pregnancies because of its steady linear growth and its independence from growth abnormalities. Its diagnostic usefulness is highlighted by the substantial connection found with GA and traditional biometric indicators. FKL can be incorporated into routine obstetric ultrasonography to improve gestational assessment accuracy and facilitate more informed clinical decision-making. Further multicentric research is necessary to confirm its wider application in high-risk pregnancies and varied demographics.
